# HEALTH INFORMATION’S IMPACT ON OSTEOPOROSIS SELF-MANAGEMENT BEHAVIORS IN OLDER WHITE AND ASIAN WOMEN

**DOI:** 10.1093/geroni/igz038.1537

**Published:** 2019-11-08

**Authors:** Yan Du, Jing Wang, Qingwen Xu

**Affiliations:** 1 The University of Texas at San Antonio, School of Nursing, San Antonio, Texas, United States; 2 School of Social Work, NY, New York, United States

## Abstract

Little is known about how health information obtained from different types of social networks affect health behaviors. This study aimed to explore the effect of health information on osteoporosis management behaviors among White and Asian women from a social capital (SC) perspective using a variety of SC measures (e.g. bonding: family, friends, coworkers; bridging: churches, clubs; linking: health providers). Semi-structured interviews were conducted with 10 White and 10 Asian women aged 50 and over in 2016. Through content analysis, we found that SC possession was different between older White and Asian women, and SC utilization to obtain health information corresponded with their possession of SC. Comparing to other diseases, health information relevant to osteoporosis was less frequently communicated. Health information from different types of SC interactively shaped participants’ behaviors. The findings suggest that culturally appropriate health interventions might improve older White and Asian women’s self-management behaviors of osteoporosis.

